# Landscape of Overlapping Gene Expression in The Equine Placenta

**DOI:** 10.3390/genes10070503

**Published:** 2019-07-02

**Authors:** Pouya Dini, Jamie Norris, Hossam El-Sheikh Ali, Shavahn C. Loux, Mariano Carossino, Alejandro Esteller-Vico, Ernest Bailey, Theodore Kalbfleisch, Peter Daels, Barry A. Ball

**Affiliations:** 1 Faculty of Veterinary Medicine, Ghent University, B-9820 Merelbeke, Belgium; 2 Gluck Equine Research Center, Department of Veterinary Science, University of Kentucky, Lexington, KY 40546, USA; 3 Theriogenology Department, Faculty of Veterinary Medicine, University of Mansoura, Mansoura City 35516, Egypt; 4 Louisiana Animal Disease Diagnostic Laboratory and Department of Pathobiological Sciences, School of Veterinary Medicine, Louisiana State University, Baton Rouge, LA 70803, USA; 5 Department of Biomedical and Diagnostic Sciences, College of Veterinary Medicine, University of Tennessee, Knoxville, TN 37996, USA; 6 Department of Biochemistry and Molecular Genetics, University of Louisville, Louisville, KY 40202, USA

**Keywords:** overlap genes, sense-antisense, different-strand overlapping transcripts, pregnancy, placenta, equine, chorioallantois

## Abstract

Increasing evidence suggests that overlapping genes are much more common in eukaryotic genomes than previously thought. These different-strand overlapping genes are potential sense–antisense (SAS) pairs, which might have regulatory effects on each other. In the present study, we identified the SAS loci in the equine genome using previously generated stranded, paired-end RNA sequencing data from the equine chorioallantois. We identified a total of 1261 overlapping loci. The ratio of the number of overlapping regions to chromosomal length was numerically higher on chromosome 11 followed by chromosomes 13 and 12. These results show that overlapping transcription is distributed throughout the equine genome, but that distributions differ for each chromosome. Next, we evaluated the expression patterns of SAS pairs during the course of gestation. The sense and antisense genes showed an overall positive correlation between the sense and antisense pairs. We further provide a list of SAS pairs with both positive and negative correlation in their expression patterns throughout gestation. This study characterizes the landscape of sense and antisense gene expression in the placenta for the first time and provides a resource that will enable researchers to elucidate the mechanisms of sense/antisense regulation during pregnancy.

## 1. Introduction 

Overlapping genes were initially thought to be only common in viruses (both DNA and RNA viruses), bacteria, mitochondria, and plasmids [1,2,3,4]. However, over the last few years, this view has changed with studies demonstrating the existence of many overlapping loci in human and murine genomes [5,6,7,8]. Since both strands of DNA are used for transcription, two main types of overlap are possible: (1) The same-strand overlap in which the two genes involved are transcribed from the same strand and (2) the opposite-strand overlap (bi-directional transcription) in which the two genes are transcribed from different strands of the same locus [6,8]. These different-strand overlapping types constitute the potential sense-antisense (SAS) gene pairs. 

The SAS gene pair is defined as two genes located on opposite genomic strands within the same locus [7,9,10]. Each antisense RNA may potentially base-pair with its complementary ‘sense’ RNA, regulating the gene expression of one another at the level of transcription, mRNA processing, splicing, or translation, among others [11,12,13,14]. Recent high-throughput transcriptome studies have revealed widespread and extensive numbers of SAS pairs in the human and murine genome [12,15,16,17,18,19]. To date, it is known that SAS pairs consist of both coding and non-coding genes, and are fundamental for the normal physiological function of cells [7,12]. Moreover, with the advances of high-throughput sequencing, it has been shown that dysregulated antisense transcript expression plays a critical role in the pathology of multiple cancers [20,21,22,23]. Currently, despite the successful assembly of the equine genome [24,25], no information is available about SAS gene expression in the equine genome. Thus, the bidirectional transcription in the equine genome needs further characterization.

Several mechanisms have been proposed for SAS interactions, including competition between antisense and sense genes over transcription factors, post-transcriptional regulation by directly blocking the binding of factors to the target transcript, or by recruiting factors that alter downstream expression [16]. These reciprocal interactions between SAS are expressed in the genome as widespread synergistic co-expression (non-random) of sense and antisense transcripts [26,27,28]. However, there are examples of SAS loci where the antisense gene downregulates expression of the sense gene [29]. In this scenario, an inverse correlation in the expression patterns of SAS is also relevant.

Currently, there is no information available on the genome wide expression patterns of SAS pairs in the mammalian placenta. In order to investigate SAS pairs expression in placenta, the fetal component of the placenta (without maternal endometrial contamination) is required. Obtaining such samples is an obstacle in species such as human and mouse with hemochorial placentation, which have a complex association between maternal and fetal cellular components [30]. In contrast to the human and mouse, the horse has an epitheliochorial placentation in which both the endometrial epithelium and the epithelium of the chorionic villi are juxtaposed with minimal extension into the uterine mucosa. Therefore, the fetal component of the placenta (chorioallantoic membrane) can be separated from the endometrium with negligible contamination by maternal cellular components. Thus, it provides an optimal model for the investigation of SAS gene expression in placentas.

Here, we hypothesized that, similar to the human and murine genome, the equine genome also harbors several SAS loci. We further hypothesized that there would be non-random positive or negative correlation in the expression patterns of SAS pairs. Stranded and paired-end RNA sequencing (RNA-seq) studies allow an unbiased genome-wide analysis of the transcriptome to elucidate the presence of SAS loci in the equine genome. Our aim was to identify the opposite-strand, overlapping genes, which are expressed in equine chorioallantois, and also to identify SAS pairs which showed negative or positive correlation in their expression patterns throughout gestation. Therefore, we utilized RNA sequencing data from equine chorioallantoic membrane (CA) [31], to identify the opposite-strand, overlapping genes. Additionally, we analyzed the expression patterns of identified SAS pairs in the chorioallantois during the course of equine gestation.

## 2. Methods

The raw read files (fastq) of a previously generated, strand-specific RNA sequencing dataset from equine chorioallantois during different stages of gestation (45 days, four, six, and ten months; four samples per time point, GSE108279) were obtained from the National Center for Biotechnology Information (NCBI) database [31]. The reads were initially trimmed for adapters and quality using TrimGalore Version 0.4.4 (Babraham Bioinformatics, Cambridge, UK). Next, the reads were mapped to the equine genome (EquCab 3.0) using STAR (Release 2.5.2b) allowing a maximum of five mismatches [32]. The mapped reads were then phased based upon the strand of transcription (termed Sense and Antisense strands) using SAMtools Version 1.3.1 [33]. Each strand was annotated (-g) using the equine reference transcriptome available in NCBI database (EquCab3.0; GCF_002863925.1, gff-spec-version 1.21, downloaded on March 2018) using Cufflinks (Release 2.2.1; http://cole-trapnell-lab.github.io/cufflinks/) [34], generating 32 samples (16 sense and 16 antisense). The start and end positions for each gene were identified and the overlap between the location of the gene in sense and antisense strands was identified using an in-house program written in java. The correlation was analyzed using Spearman correlation in JMP13 Pro statistical analysis software (SAS Institute, Cary, NC, USA), and the heatmaps were built using Package ‘*d3heatmap*’ in R [35]. Significant level was set at corrected *p*-value < 0.05 (using the Benjamini-Hochberg correction by ’p.adjust’ function in R). Gene ontology analysis performed using the protein analysis through evolutionary relationships classification system (PANTHER; Release 13.1) [36]. PANTHER classification system was used to functionally annotate genes based on gene ontology (biological process).

## 3. Results and Discussion

### 3.1. Identification and Grouping of Overlapping Genes in Placental Transcripts

**Figure 1:**
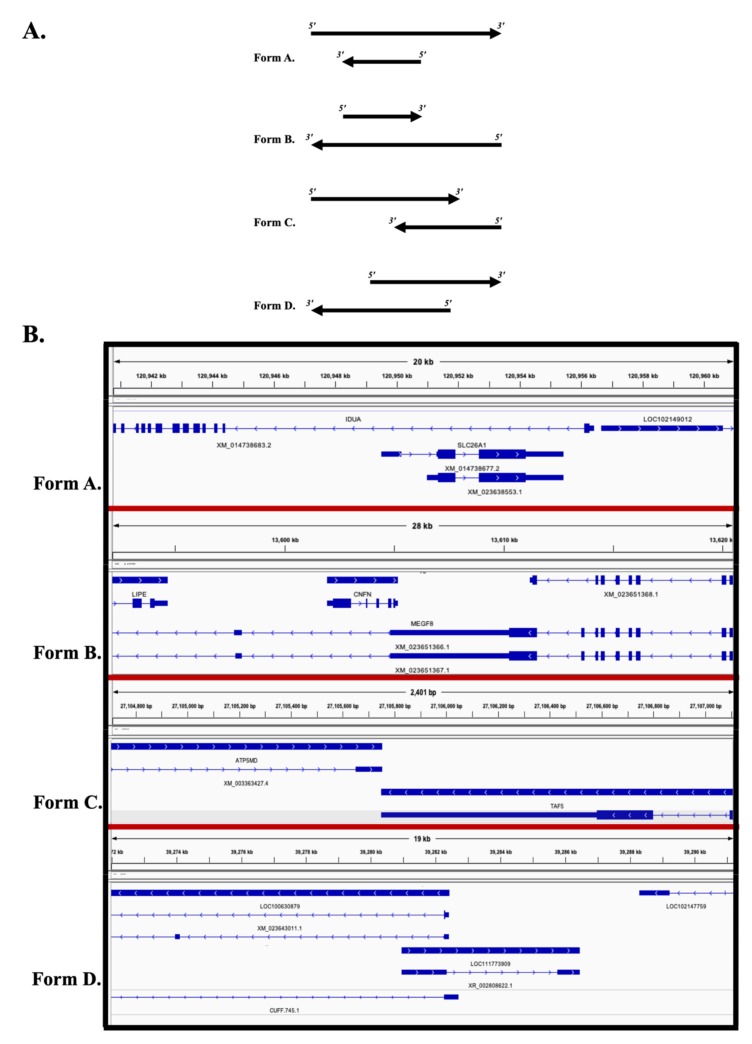
A. Schematic presentation of different forms of overlapping. (**A**) Embedded (Reverse gene is fully embedded within the Forward gene). (**B**) Embedded (Forward gene is fully embedded within the Reverse gene). (**C**) Tail-to-tail (3′-regions overlap). (**D**) Head-to-head (5′-regions overlap). B. Examples of different forms of overlapping, visualized in integrative genomics viewer (IGV). For each form, the top gene represents the sense gene and bottom gene represents the antisense gene. *IDUA* is overlapping with gene *SLC26A1*(Reverse/antisense) and fully embedded within the IDUA (Forward/sense gene), (Form A)). *CNFN* (forward/sense) is fully embedded within *MEGF8* (reverse/antisense, (Form B)). *ATP5MD* and *TAF5* has tail-to-tail overlap (Form C) and *LOC100630879* and *LOC111773909* has head-to-head overlap (Form D).

In this study, we used previously generated strand-specific RNA sequencing on 16 CA samples [31] to determine the global landscape of opposite-strand overlapping gene expression during the course of equine gestation. On average, 22.59 ± 1.3 × 10^6^ read pairs were obtained per sample (Supplementary Materials Table S1), and 91 ± 1% of the reads were uniquely mapped to the horse genome (EquCab3.0). Mapped RNA reads were phased based upon the strand of transcription and were annotated using the existing *Equus caballus* reference transcriptome (EquCab 3.0, National Center for Biotechnology Information (NCBI)) as the guide. The annotated data consist of ~30,300 genes, including protein coding genes (*n* = 21,113), lncRNA (*n* = 6787), and miRNA (*n* = 680), among others (Supplementary Materials Table S2). The overlapping of genes from the opposite strand were identified using an in-house program written in java. We further grouped the overlapping genes to four different categories: (A) Embedded (Antisense gene is fully embedded within the Sense gene); (B) embedded (Sense gene is fully embedded within the Antisense gene); (C) tail-to-tail (3′-region overlap); (D) head-to-head (5′-region overlap) (Figure 1). Using this information, we developed a bioinformatics workflow to characterize the overlapping gene expression.

### 3.2. Overlapping Gene Expression Across The Equine Transcriptome 

**Figure 2:**
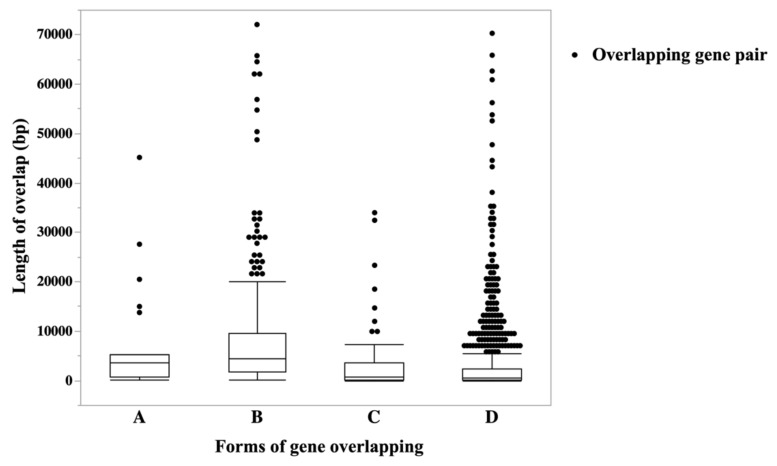
Length distribution of the overlap segments per form of gene overlapping. The overall median length of overlapping was 985 bp (1 and 72,094 bp; minimum and maximum, respectively). Each dot represents an overlapping gene pair (sense/antisense gene pairs).

A total of 1261 overlapping genes (~4% of annotated genes) were identified among all the analyzed samples (Supplementary Materials Table S3). This number is similar to the 1210 genes that represent the total number of SAS pairs expressed in the human genome [37]. In another study, a total of 615 and 497 different-strand overlapping pairs were identified in both human and mouse genomes, respectively [6]. It is noteworthy that the numbers of overlapping pairs represent approximately 10% of annotated genes. The overall median length of overlap was 985 bp (1 and 72,094 bp; minimum and maximum, respectively (Figure 2 and Supplementary Materials Table S3)). In total, 15 detected overlapping pairs had an overlap of only 1 bp. Further, we demonstrated that the head-to-head (D; ~70%) form of overlap was the most common SAS pairing, followed by embedded (A and B; ~25%) and tail-to-tail (C; ~5%) forms. This was similar to the finding of Sanna et al., in which majority of the overlap in human and murine genomes were head-to-head (D: ~50%), followed by embedded (A and B; ~29%), and tail-to-tail forms (C; ~21%) [6]. We, however, found a lower number of tail-to-tail overlap in our dataset. These analyses depend on accurate annotation of the length of the 3’ end of the gene. Annotation engines will mask regions of low complexity resulting in artificially short 3’ ends.

**Figure 3:**
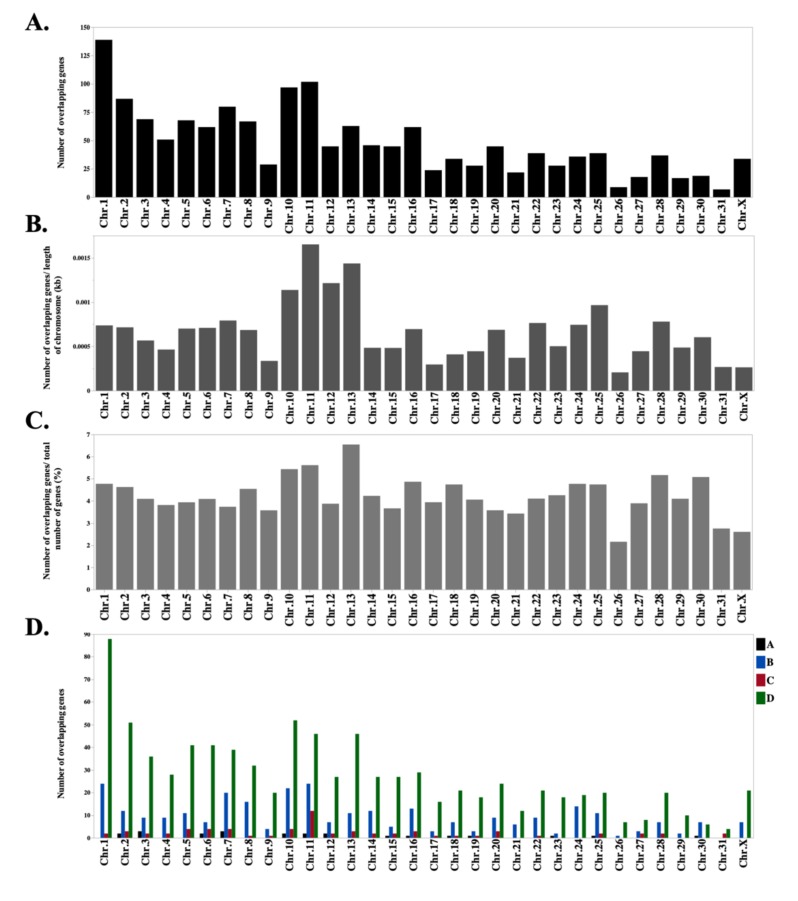
(**A**) Frequency of overlapping genes in each chromosome across the equine genome. (**B**) Number of overlapping genes normalized to the length of each chromosome in the equine genome. The number of overlapping genes was numerically higher in chromosome 11 followed by chromosomes 13 and 12; chromosome 11 harbors 7.8% of all overlapping pairs while 6.8% and 5.7% of overlapping pairs are located on chromosomes 13 and 12. (**C**) Ratio of the overlapping genes to the number of annotated genes on each chromosome. The ratio of overlapping gene was numerically higher on ECA13 followed by ECA11 and ECA10 than the other chromosomes. (**D**) Frequency of overlapping genes in each chromosome across the equine genome based on the different forms of overlapping. (A) embedded (Antisense genet is fully embedded within the Sense gene); (B) embedded (Sense gene is fully embedded within the Antisense gene); (C) tail-to-tail (3′-regions overlap); (D) head-to-head (5′-regions overlap).

The ratio of the length of overlap (bp) to the length of sense/antisense gene (bp) was calculated (Table 1). The median percentage of overlap length in relation to the length of the gene in the sense strand was 7.0% and to the gene in the antisense strand was 7.5%. To check the distribution of SAS on equine chromosomes, we further identified the number of overlapping genes on each chromosome (Figure 3A). We also normalized the number of overlapping genes on each chromosome to the length of the respective chromosome (Figure 3B). As shown in Figure 3B, the number of overlapping genes was numerically higher on chromosome 11 (ECA11), followed by ECA13 and ECA12; ECA11 harbors 7.8% of all overlapping pairs and 6.8% and 5.7% of overlapping pairs were located on ECA13 and ECA12. We further calculated the ratio (%) for the number of overlapping genes to the number of annotated genes on each chromosome (Figure 3C). The ratio of overlapping gene was numerically higher on ECA13 followed by ECA11 and ECA10 than the other chromosomes. Moreover, a similar distribution of the different forms of overlap was observed within equine chromosomes (Figure 3D). 

### 3.3. Widespread Correlation Between Sense and Antisense Gene Expression

**Figure 4:**
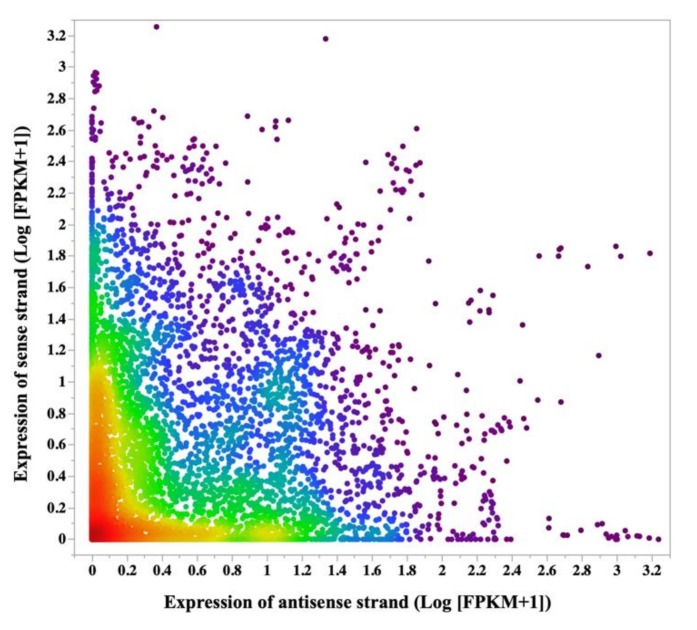
Density plot of sense and antisense strand expression. Expression in sense and antisense strands was calculated for each locus per sample. There was a numerical slight skew in the expression pattern toward antisense strand, and the sense strand showed an overall lower expression than the antisense strand (visible in the red and green dots; median of antisense expression/sense expression = 1.19). For the preparation of the density plot, the FPKMs values (Fragments Per Kilobase of transcript per Million mapped reads) were presented as Log_10_ (FPKM+1).

In the next step, we identified the biotypes of the overlapping genes in our dataset. The majority of overlapping gene interactions (SAS) were mRNA:mRNA (protein coding; 47%), followed by mRNA:lnc-RNA(~44%), and lnc-RNA:lnc-RNA (~4.7%) (Table 2). To investigate the interaction between the expression patterns of these SAS pairs, their expression patterns were analyzed throughout the course of gestation. In general, there was a numerical slight skew in the expression pattern toward antisense strand, and the sense strand showed an overall lower expression than the antisense strand (median of antisense expression/sense expression *n* = 1.19; Figure 4). A pronounced skew was observed in the gene expression pattern in human cancers, in which one strand had two to three orders of magnitude lower expression than the opposite strand [38]. The physiology behind this phenomenon need to be elucidated in future studies.

**Figure 5:**
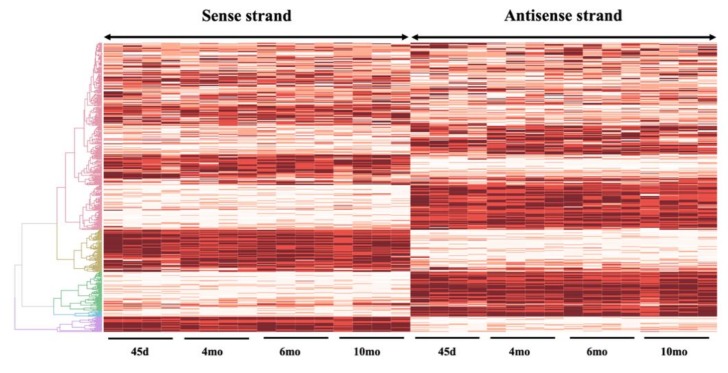
Heatmap depicting the expression of sense-antisense pairs throughout gestation in the horse. Each line represents a sense–antisense (SAS) pair; the expression values Log_10_ (FPKM+1) are indicated by color (dark red indicative of a higher expression).The heatmap was generated for the 454 genes that were expressed in at least 26 samples (32 samples in total; 16 CA samples, each divided as sense strand and antisense strand). The interactive heatmap with the possibility of focusing on the gene name along with the expression values Log_10_ (FPKM+1) can be accessed at: http://rpubs.com/pouyadini/496308.

**Figure 6:**
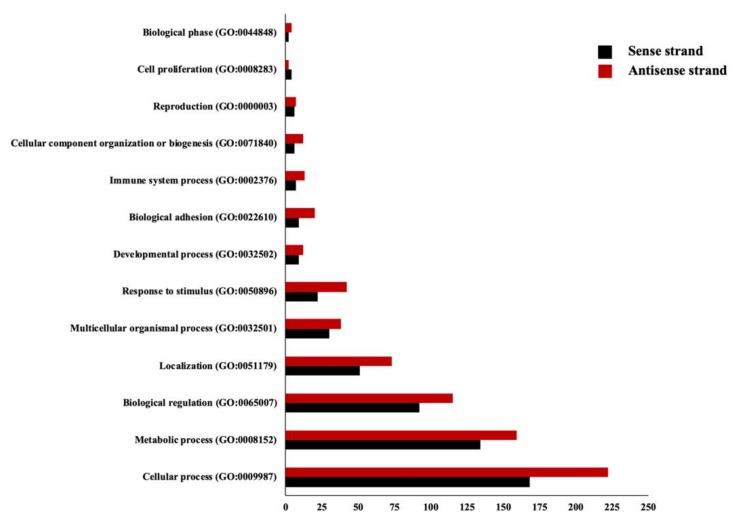
Biological functions of genes from sense and antisense strands were predicted based on gene ontology (biological process). The number of genes involved in each function is presented in the x-axis.

To demonstrate the dynamics of SAS pairs throughout pregnancy, the expression of these genes was evaluated at 45 days, four, six, and ten months of the equine gestation. The overall expression pattern of all the overlapping pairs suggested an interaction between the sense and antisense strands (Figure 5). Next, to reduce the bias in the correlation study between the sense and antisense strands, in subsequent analyses, we only proceeded with the SAS pairs, which were expressed in at least 26 samples (32 samples in total; 16 CA samples, each divided as sense strand and antisense strand). In total, 303 mRNA:mRNA, 144 mRNA:lnc-RNA, 6 lnc-RNA:lnc-RNA, and 1 mRNA:misc-RNA were used in the correlation study (454 genes in total). A systematic characterization of all sense and antisense loci expression revealed an overall positive correlation between sense/antisense genes, with an average Spearman correlation coefficient of 0.29 ± 0.21 and median of 0.24. This correlation is greater than what would be expected by chance, and was also greater than the correlation obtained between random genes on different strands [38]. This positive correlation in the expression patterns of SAS pairs is consistent with the usage of bidirectional promoters that are shared by ∼10% of protein-coding genes, which results in the co-expression of sense and antisense genes [12,31,37]. Among SAS gene pairs, expression of 34 SAS showed significant positive correlation during gestation, while eight SAS showed significant negative correlation in their expression patterns (Table 3). We further performed gene ontology analysis of genes from sense and antisense strand using the protein analysis through evolutionary relationships classification system (PANTHER; Release 13.1) [36]. In general, our predicted biological process analysis demonstrated that genes from both sense and antisense strands were involved in similar processes, with cellular and metabolic processes being the most represented (Figure 6). This is indicative of the importance of these overlap regions in the normal function of cells and tissues. The purpose of this study was to identify the SAS pairs in the equine genome as expressed in equine chorioallantois. This study constitutes a comprehensive assessment of transcription originating from the sense and antisense expression in the fetal placenta. The physiological function of these SAS pairs remains to be elucidated in future studies. 

## 4. Conclusions

Overall, our study contributes to a growing body of literature related to the presence of opposite-strand gene transcription by providing a list of overlapping loci in the equine genome. Furthermore, this study characterizes the landscape of SAS expression in equine pregnancy and provides additional information regarding the interaction between sense and antisense genes throughout gestation. This study will provide a resource that will enable researchers to elucidate the mechanisms of sense/antisense regulation during pregnancy.

## Figures and Tables

**Table 1:** The length of overlap in relation to the length of the sense and antisense genes. The degree of overlap between sense and antisense genes is expressed as percentage of the overlap length.

	Sense			Antisense		
Group	Median (%)	Min (%)	Max (%)	Median (%)	Min (%)	Max (%)
A * (*n* = 23)	8.4	0.01	81.8	100	100	100
B (*n* = 297)	100	100	100	9.6	0.06	87.8
C (*n* = 66)	4.7	<0.01	98.3	5.9	<0.01	93.6
D (*n* = 875)	3.1	<0.01	99.8	3.5	<0.01	99.9

* (A) embedded (Antisense gene is fully embedded within the Sense gene) and 100% of the length of the antisense genes are embedded in the sense gene; (B) embedded (Sense gene is fully embedded within the Antisense gene) and 100% of the length of the sense genes are embedded in the antisense gene; (C) tail-to-tail (3′-regions overlap); (D) head-to-head (5′-regions overlap).

**Table 2:** Biotypes of the overlapping genes in the dataset distributed across the overlapping forms.

Biotypes	A *	B	C	D
mRNA:mRNA (47%)	7	42	29	520
mRNA:lnc-RNA (44%)	12	205	31	309
lnc-RNA:lnc-RNA (4.7%)	1	17	4	37
mRNA:miRNA (2.6%)	2	23	-	1
lnc-RNA:tRNA (0.5%)	-	-	3	4
miRNA:miRNA (0.3%)	-	4	-	-
lnc-RNA:miRNA (0.3%)	-	4	-	-
mRNA:miscRNA (0.2%)	-	1	-	2
mRNA:snRNA (0.07%)	-	-	-	1
lnc-RNA:snRNA (0.07%)	1	-	-	-
lnc-RNA:miscRNA (0.07%)	-	-	-	1

* (A) embedded (Antisense gene is fully embedded within the Sense gene); (B) embedded (Sense gene is fully embedded within the Antisense gene); (C) tail-to-tail (3′-regions overlap); (D) head-to-head (5′-regions overlap).

**Table 3:** List of sense and antisense pairs that showed a significant correlation in their expression pattern between the sense and antisense genes (*n* = 16 pairs of sense and antisense transcriptome).

Sense	Antisense	Spearman Correlation	Corrected *p*-Value
*NPHP3*	*UBA5*	0.9112	0.000001
*MSMP*	*RGP1*	0.9001	0.0057
*PSPC1*	*MPHOSPH8*	0.8778	0.0042
*LOC100059263*	*PGRMC1*	0.8535	0.000001
*LYRM2*	*ANKRD6*	0.8392	0.0367
*LOC102147537*	*THAP1*	0.7845	0.0009
*NDRG2*	*SLC39A2*	0.7728	0.0004
*GLS2*	*SPRYD4*	0.7155	0.0018
*EML6*	*RTN4*	0.7105	0.002
*KIAA0895*	*ANLN*	0.7054	0.0071
*TAF10*	*ILK*	0.6946	0.0028
*LOC111775727*	*C11H17orf49*	0.6933	0.0262
*LOC111774163*	*CNN2*	0.6813	0.0037
*TUBGCP6*	*SELENOO*	0.6793	0.0038
*P2RY11*	*EIF3G*	0.6681	0.0047
*LOC106782172*	*MTO1*	0.6673	0.0047
*TMEM259*	*GRIN3B*	0.6500	0.0064
*LOC100063824*	*CCDC51*	0.6477	0.0429
*PPP2R3B*	*LOC102148365*	0.6429	0.0072
*RABEP2*	*ATP2A1*	0.6286	0.0091
*MYO19*	*ZNHIT3*	0.6236	0.0098
*BCCIP*	*DHX32*	0.6035	0.0647
*ENTPD8*	*NOXA1*	0.5865	0.0169
*ASRGL1*	*LOC111775953*	0.5862	0.0749
*MYO15B*	*LOC111775588*	0.5622	0.0719
*CIDEB*	*NOP9*	0.5493	0.0275
*LOC111772592*	*ELOVL6*	0.5475	0.0281
*LOC106783384*	*TNPO2*	0.5469	0.043
*NR2C2AP*	*RFXANK*	0.5271	0.0359
*TMCO6*	*LOC111767785*	0.5184	0.0396
*GNE*	*CLTA*	0.5119	0.0427
*TNFRSF17*	*LOC111767561*	0.5001	0.0576
*LOC100053030*	*GAS8*	0.4987	0.0493
*STAT2*	*IL23A*	0.4985	0.0494
*BAK1*	*LOC111769307*	−0.5034	0.0468
*MYBBP1A*	*SPNS2*	−0.5172	0.0402
*AHSA2*	*USP34*	−0.5227	0.0456
*LOC111773182*	*LRRC72*	−0.6117	0.0455
*PCYT1B*	*PDK3*	−0.6362	0.048
*IGSF10*	*MED12L*	−0.6552	0.008
*CD72*	*TESK1*	−0.7735	0.0012
*LOC111774293*	*KRI1*	−0.7918	0.0192

## Supplementary Materials

The following are available online at https://www.mdpi.com/2073-4425/10/7/503/s1, Table S1: Read counts and mapping quality of Illumina RNA-sequencing dataset from placenta at different stages of equine pregnancy (GSE108279; mapped to Ecab3.0), Table S2: The list of annotated genes and their expression values (FPKM) for forward and reverse genes in CA at different stages of pregnancy, Table S3: The list of all overlapping genes along with the forms of overlapping and their biotypes.
